# Motor Improvement in Adolescents Affected by Ataxia Secondary to Acquired Brain Injury: A Pilot Study

**DOI:** 10.1155/2019/8967138

**Published:** 2019-11-26

**Authors:** Elisabetta Peri, Daniele Panzeri, Elena Beretta, Gianluigi Reni, Sandra Strazzer, Emilia Biffi

**Affiliations:** Scientific Institute IRCCS Eugenio Medea, Bosisio Parini, Lecco, Italy

## Abstract

**Aim:**

To assess changes in locomotion and balance in adolescents affected by ataxia secondary to acquired brain injury after a rehabilitation treatment with physiotherapy and the Gait Real-time Analysis Interactive Lab (GRAIL), an immersive virtual reality platform.

**Methods:**

11 ataxic adolescents (16(5) years old, 4.7(6.7) years from injury) underwent 20 45-minute sessions with GRAIL plus 20 45-minute sessions of physiotherapy in one month. Patients were assessed before and after rehabilitation with functional scales and three-dimensional multiple-step gait analysis.

**Results:**

Results showed significant improvements in ataxia score assessed by the Scale for the Assessment and Rating of Ataxia, in dimension D and E of Gross Motor Function Measure, in walking endurance and in balance abilities. Moreover, the training fostered significant changes at hip, knee, and ankle joints, and the decrease of gait variability, toward healthy references.

**Interpretation:**

In spite of the pilot nature of the study, data suggest that training with immersive virtual reality and physiotherapy is a promising approach for ataxic gait rehabilitation, even in chronic conditions.

## 1. Introduction

Ataxia is a neurological sign resulting in cognitive and motor deficits that have a significant impact on quality of daily life. The locomotion ability is impaired by decreased balance due to loss of coordination, dysmetria, tremors, and hypotonia [1]. Cerebellar ataxia is a form of ataxia that originates from a deficit located in the cerebellum [2]. Its etiology is either genetic or it is a consequence of an acquired brain injury (ABI). In the latter case, it can be due to a focal lesion as a result of hemorrhagic events, traumatic brain injury or brain tumor, often located in the posterior cranial fossa. Ataxia in children and adolescents usually entails a relevant social and economic burden, since it involves young subjects that may need assistance and restorative treatments to be paid by the community throughout all life.

Cerebellar ataxic gait shows some common clinical signs: a generalized irregular gait pattern, reduced gait speed and cadence, shorter step and stride length, reduced swing phase, increase of the step width[3], augmented stance phase and double limb support time [4], increased variability in timing [1, 5], knee hyperextension, reduced power at the ankle joint, loss of smoothness during the gait, and decomposition of multijoint movements into a series of single-joint movements [6]. These signs can increase the exposure to fall risk, reduce the endurance during the walk and, in more severe cases, require the use of orthosis, limiting a complete access to peer activities.

Therefore, the recovery of locomotion is commonly considered one of the primary goals in rehabilitation of ataxic patients.

Previous studies described motor recovery [7] and reported successful outcome in patients with degenerative ataxia [8–11], while only few low-level evidence publications tackle the rehabilitation of patients with ataxia secondary to ABI [7].

Two main rehabilitative approaches are usually considered [12], named as restorative and compensatory strategy. The former aims at restoring the functional ability through the recovery of the neuro-musculoskeletal system, while the latter consists of a series of strategies that the patient has to learn to get access to a determinate function.

The choice is guided by the distance from the injury: a very early intervention exploits the plasticity mechanisms and the subject has a better chance to regain functions working with a restorative approach, trying to re-learn the more correct patterns. In contrast, when the distance from the injury increases and the residual potential relearning declines, the compensatory approach is usually preferred.

In the last years, the advent of advanced technologies in rehabilitation and, specifically, of Virtual Reality (VR)-based devices, has unfolded new possibilities in the world of rehabilitation [13–16]. VR is a promising strategy that incorporates many principles recognized as crucial for motor relearning, such as high intensity, repetitive and goal-oriented tasks, enhanced synchronized sensorial cues and active participation [17–19]. Furthermore, the combination of biofeedback has already shown to be effective [20]. The virtual environment is suitable for a tailor-made work too, as it can be adapted to the individual needs of each patient.

Platforms integrating immersive VR (IVR) with an instrumented treadmill and a motion capture system combine assessment and rehabilitation purposes. Instrumented treadmills are able to acquire gait data recording many consecutive steps, differently to traditional gait analysis devices. This allows to evaluate the step variability, which is particularly relevant in the ataxic population [21], who exhibits an increased variability in terms of step length, excursion, and timing of hip, knee, and ankle joints [5].

VR training with these kinds of platforms has been previously studied on children affected by Cerebral Palsy (CP) [22] and with ABI [23].

Since there are no studies about the rehabilitation of ataxic children and adolescents by means of similar platforms, the aim of this work is to investigate the potential of the use of IVR, biofeedback, and treadmill walking combined with physiotherapy, in this population and to highlight the advantages given by the assessment of the multistep gait analysis in ataxic subjects.

## 2. Methods

### 2.1. Participants

Patients affected by ABI who exhibited ataxia secondary to trauma, brain tumor, stroke, encephalitis, anoxia, or arteriovenous malformation were recruited at the Scientific Institute Medea. The inclusion criteria were: signs of ataxia, identified by clinical assessment; age between 7 and 30; mild to moderate gross motor ability–level I–III of Gross Motor Function Classification System (GMFCS) [24, 25]; compliance and ability to understand and execute test instructions. The exclusion criteria were: severe muscle spasticity, a diagnosis of severe learning disability or behavioral problems, and visual difficultiesthat would impact on function and participation.

The protocol was approved by the ethics committee of Scientific Institute Medea and conducted in accordance with the Declaration of Helsinki. Patients or their parents subscribed a written informed consent.

### 2.2. Gait Real-Time Analysis Interactive Lab

**Figure 1:**
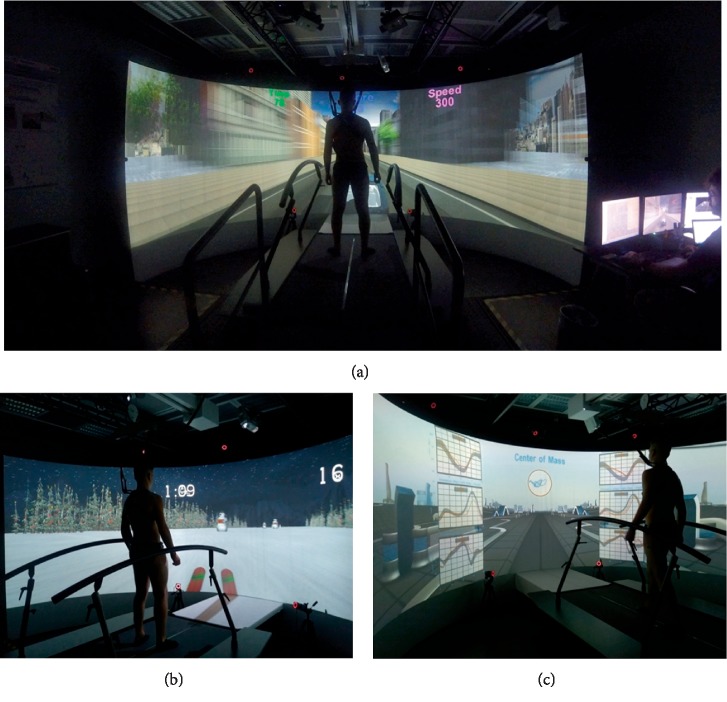
(a) The GRAIL system. (b) Example of balance training exercise: the subject performs a slalom on the snow by shifting the body weight left and right. (c) Example of exercise to train locomotion and gait pattern: the kinetics and kinematics of joints during walking are projected in real time as feedback.

Medea is equipped with an IVR system, the Gait Real-time Analysis Interactive Lab (GRAIL) by Motekforce Link (the Netherlands). This device integrates IVR with an instrumented treadmill and a motion capture system, that can be used for rehabilitation and assessment purposes. It includes a semicircular screen where the virtual reality environment is projected and a dual-belt treadmill, which integrates two force platforms and is synchronized with the projected environment. To assure a safe use, a harness and two lateral handrails are used (Figure 1(a)).

As a rehabilitative tool, the GRAIL provides biofeedback based on kinematic or kinetic gait features, allowing to train left and right body side thanks to the split-belt treadmill, and to coach balance and coordination control.

As assessment tool, the GRAIL is equipped with 10 optoelectronic cameras and three video cameras that, together with the two force platforms, can be used to acquire spatial, temporal, kinematic, and kinetic parameters of many consecutive steps in real time. These data can also be exploited as visual feedback to the operator and patient during the training.

### 2.3. Study Design and Intervention

Patients underwent 20 45-minute sessions of training with GRAIL plus 20 45-minute sessions of physiotherapy within one month. The therapeutic scheme was customized over the patients' need, tailoring the setting and the difficulty of proposed exergames on patients' skills, and it was oriented to the recovery of balance ability and of a correct locomotion pattern.

The balance training encompassed left-right shifting of body weight, monopodalic support, or balance maintenance while receiving external swinging stimuli (Figure 1(b)).

The gait training included gradually increasing difficulty exercises, from the control of the center of mass during walking without the upper limb support to more challenging tasks, like multitasking activities during walking, external perturbations (i.e., changes of treadmill slope, single belt sliding, medio-lateral belt sways) and locomotion with decreased step width (i.e., one belt only activated). The kinetics and kinematics of target districts were projected in real time as feedback and overlaid to healthy reference values (Figure 1(c)).

Concerning physiotherapy, exercises were aimed at reinforcing the activities trained with VR, focusing on monopodalic balance training, walking on narrow path, walking on irregular path, get up and down the stairs, jump and run.

### 2.4. Outcome Measures

Patients' performance was evaluated before (T0) and after (T1) rehabilitation by means of functional scales and three-dimensional gait analysis.

The Scale for the Assessment and Rating of Ataxia (SARA) is a scale developed to quantify the severity of the ataxia from 0 (no ataxia) to 40 (severe ataxia). It includes motor tasks (that investigate the most common deficits, like imbalance, tremors, dysmetria, and rhythmic movements) and speech [26].

The gross motor ability of patients was assessed by means of Gross Motor Function Measure (GMFM-88): it ranges from 0 (severe deficit) to 100 and is composed of 88 items divided into 5 sections: A–lying and rolling; B–sitting; C–crawling and kneeling; D–standing; E–walking, running, and jumping [27].

The six-minute walking test (6MWT) evaluated walking endurance [28], measuring the distance covered over six minutes of self-paced walking along a standardized path.

The Berg Balance Scale (BBS) assesses patients' fall risk and was used to evaluate patient's balance ability [29]. BBS is composed of 14 items scored with a five-point scale (0–4) according to functional level. The total score ranges from 0 (high fall risks) to 56 (low fall risks).

The 3D gait analysis (GA) was acquired on the GRAIL. Subjects were asked to walk wearing socks for an adaptation period of six minutes on the instrumented treadmill at a fixed velocity, customized over the patient's ability. Then, about 20 steps were acquired. During this assessment no biofeedback was provided with the GRAIL.

At the end of the treatment, qualitative improvements of each patient in terms of activities and participation were collected by patients and their families.

### 2.5. Data Analysis

Concerning GA data, the Gait Offline Analysis Tool was used to load the .mox file, to filter data (2^nd^ order Butterworth filter, cut off frequency at 6 Hz), to exclude strides with misplaced feet (e.g., foot on both belts or on the opposite belt) and to export the kinematics and kinetics traces in a .csv file. The gait parameters were then computed with an ad-hoc MATLAB (The MathWorks®) software that, for each step, extracted gait features at hip, knee, and ankle levels for left and right side. Since ataxia is not characterized by laterality, the mean value between the right and left parameters was considered.

The gait parameters computed were: the stance period, computed as the percentage ratio between the stance phase (from the initial contact of foot and the toe off of the same limb) and the gait cycle, the step length, the step width, and the gait speed as spatio-temporal parameters; the peak flexion power for the ankle, knee, and hip joints for kinetic evaluations; the peak of flexion and extension for the three joints in the sagittal plane; the range of motion (ROM) of hip abduction/adduction, and the ROM of pelvis in the three planes (tilt, obliquity and rotation).

To evaluate the variability of gait, for each parameter the coefficient of variation (CV) was computed as the percentage ratio between the SD and the mean of the steps of each *i*-th subject [30], as in Equation (1).

(1) $$\begin{array}{c} {\text{CV}}_{i}=\frac{{\text{SD}}_{i}}{{\text{mean}}_{i}}100. \end{array}$$

The coefficient of variation is not appropriate for negative datasets or with values around zero [31]. For parameters that assumed values ≤0, the SD has been reported.

The normality of the data was checked with the Shapiro–Wilk test. Since not all the measures were normal, nonparametric statistical analysis was carried out; medians and interquartile ranges are reported.

A within-group comparison was performed by means of the Wilcoxon test.

To help the interpretation of GA, patients outcomes were compared with those obtained by a healthy control group of 16 subjects (mean (SD) age of 10.0 (1.3) years, 15 males). These data were included in a previous study on autism [32]. The between-group comparison of GA outcomes was performed with Mann–Whitney *U*-test.

The analysis was carried out with IBM SPSS Statistics v15 and the significance level was set at 5%.

## 3. Results

According to the inclusion criteria, 11 subjects (age from 9 to 27) were recruited for the study. Demographic characteristics are summarized in Table 1. Accidentally, only patients with GMFCS level of II agreed to participate.

Table 2 shows the results in terms of functional scales.

The signs of ataxia significantly diminished across the training, with a reduction of 20% of the SARA. GMFM-88, and its subscales, 6MWT and balance ability showed significant improvements after the treatment.

Table 3 summarizes results obtained in terms of GA.

A significant variation of step length, gait speed, maximal ankle power, maximal degree of knee flexion, and ROM of hip during abduction was obtained over time. Furthermore, the significant differences observed with respect to the healthy control group in terms of step length, gait speed, maximal ankle power, maximal knee extension and ROM of pelvic rotation disappeared after treatment.

Finally, the CV of step length, gait speed, maximal ankle power, and ROM of pelvic obliquity and rotation significantly reduced after therapy. This was supported by an overall trend of reduction of CV in almost all the parameters.

All the data were collected for the whole group.

Concerning qualitative changes of each patient in terms of activities and participation, improvements for 9 patients out of 11 were observed. Specifically, three patients had significant improvement in terms of safety during activities in standing and walking; three patients, that had reported difficulties in dual task activities such as walking and speaking before the treatment, enhanced their ability to fix a trajectory and keep balance while interacting with other people; one patient was able to go out alone, using public transports after the treatment; two patients showed pain decrease due to the improvement of their gait pattern.

## 4. Discussion

Children and adolescents with ataxia exhibit coordinative limitations, which often affect their locomotion and balance. In recent years, more and more attention has been devoted to training that exploit the potential of VR in rehabilitative context, but up to now only few studies described improvements on children with ABI treated with VR [23, 33], and, to our knowledge, none specifically on ataxic patients.

This manuscript aims at investigating the feasibility and effectiveness of a rehabilitation program that exploits IVR, biofeedback, and treadmill walking coupled to physiotherapy for the recovery of locomotion and balance in adolescents affected by ataxia secondary to ABI.

The study showed that the training was feasible and well tolerated by patients. No subject withdrew from the study, and patients and their families gave positive feedbacks in terms of engagement and functional recovery.

Our findings support that 20 sessions of treadmill training augmented by IVR together with 20 sessions of physiotherapy are effective for a significant improvement of balance and locomotion functions in ataxic adolescents. The ataxia level was significantly diminished, the gross motor ability was significantly improved in terms of standing and walking, and the balance and the walking endurance improved as well.

In terms of GA, subjects showed at T0 reduced gait speed, shorter step length and swing phase, diminished maximal ankle power, reduced knee flexion, and a generalized increase in step variability, with respect to their healthy pairs. These gait features well match with the typical clinical signs of cerebellar ataxic gait [3–6].

Data obtained after the proposed treatment showed improvements towards the pattern of typically developing subjects in terms of step length, gait speed, kinetic of the movement at ankle, and kinematic of knee, hip and pelvic joints. Evidence of reduction of the variability among steps was also obtained, suggesting the achievement of a more regular gait pattern. Furthermore, a reduction of step width, even if not significant, was observed and the two patients that were affected by knee hyperextension showed improvements after the combination of IVR and physiotherapy treatment.

Comparison among our results and what was previously reported is limited by the paucity and low-level of evidence reported on ataxia [7]. Previous case-studies have shown some improvements induced by treadmill training or virtual exergames on ataxic adults. Three case reports evaluated five adult ataxic patients (three secondary to traumatic brain injury and two secondary to tumor resection) who underwent trunk exercises and treadmill training with body weight support (15 and 30 sessions). The participants obtained some improvements in terms of BBS and gait [34–36]. Differently, no evidence of improvements induced by a four-week training with VR exergames on five ataxic adults have been reported in terms of balance ability and gait parameters, although SARA highlighted progresses [37].

The effectiveness of IVR treadmill training plus physiotherapy on ataxic adolescents has never been investigated. However, its performance in young patients with ABI has been previously studied [23], with comparable improvements in terms of gross motor abilities (GMFM-D improved of 6.6% in ABI and of 5.4% in ataxic patients; GMFM-E improved of 11.5% in ABI and of 4.4% in ataxic patients), but smaller ones in terms of 6MWT (improvements of 60.0% in ABI and of 9.6% in ataxic subjects). However the different mean value at baseline (277 m vs 500 m in the current work) may have limited the improvements due to ceiling effect (95% of confidence interval in age-matched healthy population is 651–742 m [38]).

Modifications induced by training based on IVR and physiotherapy look effective in modifying both the locomotion pattern as a whole and the control of the local districts in ataxic patients. Indeed, the present work supports that the treatment induces a modification of the gait kinematics. With respect to other technologically-advanced tools, such as robot-assisted gait training applied in the context of ABI rehabilitation, the effects obtained combining physiotherapy with IVR treadmill training seems to be particularly promising at ankle and knee levels. Indeed, a previous study on 23 children with ABI showed improvements induced by 20 sessions of robot-assisted gait training in terms of gross motor abilities, walking endurance and gait kinematics at hip level, while no changes were observed for more distal districts [39].

One of the strengths of the present work is that we exploited the GRAIL technology to quantitatively analyze the variability of the locomotion, which is a crucial aspect of ataxic patients. Their high gait irregularity prevents from obtaining reliable results by using standard gait analysis, during which the user usually selects one or few steps that should represent the gait pattern of a single patient. Due to the specificity of ataxia, it is mandatory to use a multistep approach to quantify ataxic locomotion. Our results, based on the multistep gait analysis available through GRAIL, give encouraging evidence of reduction of step variability, toward the pattern of healthy individuals.

A limit of this work is that, although GMFCS has been used in the past for the description of patients with ABI [25], we are not aware of any systematic validation in children with this pathology. Furthermore, the increased effectiveness of a combined IVR treadmill training plus physiotherapy with respect to traditional approaches has to be deepened in the future by means of randomized controlled trials. The generalizability of the results of the present work is currently limited by the little sample size, the heterogeneity of patients in terms of age, and the absence of a control group.

However, it is noteworthy that the population analyzed had a median (interquartile) distance from trauma of 4.7 (6.7) years. As studied by Kuper and colleagues, the spontaneous recovery of balance ability in children after cerebellar tumor resection is relevant over the first three months post-surgery and continues over the first year post surgery [40]. The recovery of the remaining motor ability shown in the present work may thus be ascribed to the rehabilitation treatment.

## 5. Conclusion

To conclude, the present study shows the first evidence of the effectiveness of immersive virtual reality treadmill training together with standard physiotherapy on ataxic adolescents. The intervention proposed was customized on patients' need; it was ecological and highly motivating. Forty sessions of such training produced significant improvements in locomotion pattern, balance, and reduction of gait variability, towards healthy references. Finally, although specific goals in terms of activity and participation were not defined, the proposed protocol improved patients' participation to social life, as reported both by patients and their parents.

**What This Paper Adds**

- Assessment of preliminary effectiveness of IVR devices in the ataxic patient rehabilitation.
- Modifiability of locomotion and balance of ataxic adolescents, even in chronic condition.
- Reliable evaluation of gait pattern and step variability in ataxic population.

## Figures and Tables

**Table 1:** Demographic features of the patients included into the study.

Age (y)	16.0 (5.0)
Gender (M/F)	6/5
Time from injury (y)	4.7 (6.7)
Etiology (tumor/AVM/TBI)	9/1/1
GMFCS (I/II/III)	0/11/0

Data are reported as median (interquartile ranges). M: male; F: female; y: years; AVM: arteriovenous malformation; TBI: traumatic brain injury; GMFCS: gross motor function classification system.

**Table 2:** Results of functional scales before (T0) and after (T1) training.

	T0	T1	*p*-value
SARA	10.5 (4.5)	8.5 (2.8)	0.012
GMFM-88	97.0 (4.0)	98.0 (2.5)	0.004
GMFM-D	92.0 (4.0)	97.0 (3.5)	0.008
GMFM-E	90.0 (14.0)	94.0 (7.5)	0.001
6MWT	500 (96)	548 (100)	0.005
BBS	53.0 (3.5)	54.0 (2.0)	0.016

Data are reported as median (interquartile ranges). *p*-values refer to the non parametric paired Wilcoxon test. SARA: Scale for the assessment and rating of ataxia; GMFM-88: gross motor function measure expressed as percentage, dimension D (standing) and E (walking); 6MWT: six minute walking test; BBS: Berg balance scale.

**Table 3:** Temporal, spatial, kinematics and kinetics characteristics acquired with gait analysis on GRAIL before (T0) and after (T1) training.

	T0	T1	Healthy	*p*-val T0 vs T1	*p*-val T0 vs healthy	*p*-val T1 vs healthy
Stance%	69.4 (1.7)	69.1 (2.1)	67 (2.5)	0.123	*0.001*	*0.008*
CV%	4 (1.6)	3 (1.8)	2.1 (1.3)	0.175	*0.013*	0.094
Step length [cm]	36.6 (2.5)	42.7 (8)	42.1 (10)	*0.001*	*0.019*	0.863
CV%	12 (3.6)	9.4 (3.4)	7.5 (2.3)	*0.001*	*0.004*	0.225
Step width [cm]	18.9 (7)	16.6 (4.1)	15 (5)	0.206	0.190	0.387
CV%	16.9 (6.2)	19.6 (8.9)	14 (5)	0.147	0.226	0.132
Gait speed [cm/s]	66.9 (10.7)	78.1 (12.1)	88.4 (24.9)	*0.001*	*0.000*	0.057
CV%	10.2 (4.8)	8.5 (3.2)	5.4 (1.1)	*0.019*	*0.000*	*0.000*
Max ankle power [W]	0.9 (0.2)	1.3 (0.5)	1.3 (1.3)	*0.020*	*0.008*	0.477
CV%	31.5 (15)	24.1 (8.5)	28.2 (12.4)	*0.002*	0.215	0.692
Max knee power [W]	0.6 (0.3)	0.6 (0.3)	0.9 (0.3)	0.322	*0.003*	*0.014*
CV%	27 (12.9)	26.1 (7.4)	29.8 (13.6)	0.492	0.895	0.732
Max hip power [W]	0.4 (0.2)	0.5 (0.1)	0.9 (0.4)	0.105	*0.001*	*0.016*
CV%	25.3 (8.7)	23.1 (7.6)	20.8 (9.3)	0.193	0.179	0.895
Max ankle flex [°]	11.9 (2.3)	13.9 (3.1)	17.6 (2.7)	0.067	*0.001*	*0.002*
CV%	11.2 (7.5)	12.3 (7)	7.3 (4.3)	0.520	*0.019*	*0.021*
Max ankle ext [°]	6.1 (3.5)	6.7 (4.9)	5.4 (4.7)	0.577	0.711	0.289
SD∗	2.2 (1.2)	2.3 (1)	2.9 (2.5)	0.898	0.474	0.474
Max knee flex [°]	57 (6.8)	59 (5.2)	63.2 (5)	*0.032*	*0.001*	*0.005*
CV%	6.2 (2.9)	6.2 (2.2)	3.8 (1.7)	0.240	*0.004*	*0.007*
Max knee ext [°]	−3.1 (3.4)	−3 (4.3)	0.1 (4.4)	0.966	*0.025*	0.051
SD∗	2.2 (0.5)	1.9 (0.8)	1.5 (1.1)	0.966	0.160	0.289
Max hip flex [°]	33.3 (7.3)	35.3 (9.4)	32.4 (10.2)	0.278	0.786	0.388
CV%	6 (2.3)	5.8 (1.5)	5.5 (2.3)	0.638	0.245	0.473
Max hip ext [°]	2.2 (7.5)	1.2 (7.8)	3.4 (9.6)	0.413	0.336	0.388
SD∗	2.6 (1.1)	2.5 (1.1)	2.1 (0.8)	0.700	0.098	0.160
ROM hip abd [°]	9.2 (4.3)	11.9 (4)	11.7 (4)	*0.001*	0.145	0.863
CV%	16.9 (7.9)	17 (3.9)	15.3 (1.9)	0.365	0.097	0.287
ROM pelvic tilt [°]	4.9 (0.9)	5.2 (1.8)	4.2 (1.2)	0.102	*0.022*	*0.005*
CV%	30.2 (4.5)	28.7 (3.1)	26 (6.2)	0.240	*0.040*	0.174
ROM pelvic ob [°]	5.7 (2.1)	6.4 (1.9)	6.6 (2.4)	0.413	0.604	0.980
CV%	23.7 (4.8)	18.3 (7.2)	17.7 (4.8)	*0.010*	*0.006*	0.415
ROM pelvic rot [°]	10.8 (4.4)	11.5 (4.7)	8.4 (5.1)	0.175	0.120	*0.013*
CV%	28.1 (9.1)	28.5 (6.6)	28.8 (8.9)	*0.042*	0.711	0.748

Data are reported as median (interquartile ranges); CV: coefficient of variation; SD: standard deviations; ROM: range of motion; flex: flexion; ext: extension; *p*-val: *p* values. *P* values refer to the nonparametric paired Wilcoxon test in column “T0 vs T1”, while they refer to the nonparametric Mann–Whitney *U*-test in column “T0 vs healthy” and “T1 vs healthy”.

## Data Availability

The datasets used and/or analysed during the current study are available from the corresponding author on reasonable request.

## Abbreviations

VR: Virtual reality
GMFCS: Gross motor function classification system
GRAIL: Gait real-time analysis interactive lab
SARA: Scale for the assessment and rating of ataxia
GMFM-88: Gross motor function measure-88 items
6MWT: Six minute walking test
BBS: Berg balance scale
GA: Gait analysis
ROM: Range of motion
CV: Coefficient of variation
AVM: Arteriovenous malformation
TBI: Traumatic brain injury
SD: Standard deviations.
